# XRCC1 Gene Polymorphisms and Glioma Risk in Chinese Population: A Meta-Analysis

**DOI:** 10.1371/journal.pone.0111981

**Published:** 2014-11-06

**Authors:** Li-Wen He, Rong Shi, Lei Jiang, Ye Zeng, Wen-Li Ma, Jue-Yu Zhou

**Affiliations:** 1 Institute of Genetic Engineering, Southern Medical University, Guangzhou, China; 2 Zhujiang Hospital, Southern Medical University, Guangzhou, China; 3 Department of Neurosurgery, Changzheng Hospital, Second Military Medical University, Shanghai, China; 4 Department of Stomatology, Nanfang Hospital, Southern Medical University, Guangzhou, China; Sudbury Regional Hospital, Canada

## Abstract

**Background:**

Three extensively investigated polymorphisms (Arg399Gln, Arg194Trp, and Arg280His) in the X-ray repair cross-complementing group 1 (XRCC1) gene have been implicated in risk for glioma. However, the results from different studies remain inconsistent. To clarify these conflicts, we performed a quantitative synthesis of the evidence to elucidate these associations in the Chinese population.

**Methods:**

Data were extracted from PubMed and EMBASE, with the last search up to August 21, 2014. Meta-analysis was performed by critically reviewing 8 studies for Arg399Gln (3062 cases and 3362 controls), 8 studies for Arg194Trp (3419 cases and 3680 controls), and 5 studies for Arg280His (2234 cases and 2380 controls). All of the statistical analyses were performed using the software program, STATA (version 11.0).

**Results:**

Our analysis suggested that both Arg399Gln and Arg194Trp polymorphisms were significantly associated with increased risk of glioma (for Arg399Gln polymorphism: Gln/Gln vs. Arg/Arg, OR = 1.82, 95% CI = 1.46–2.27, *P* = 0.000; Arg/Gln vs. Arg/Arg, OR = 1.25, 95% CI = 1.10–1.42, *P* = 0.001 and for Arg194Trp polymorphism: recessive model, OR = 1.78, 95% CI = 1.44–2.19, *P* = 0.000), whereas the Arg280His polymorphism had no influence on the susceptibility to glioma in a Chinese population.

**Conclusions:**

This meta-analysis suggests that there may be no association between the Arg280His polymorphism and glioma risk, whereas the Arg399Gln/Arg194Trp polymorphisms may contribute to genetic susceptibility to glioma in the Chinese population. Nevertheless, large-scale, well-designed and population-based studies are needed to further evaluate gene-gene and gene–environment interactions, as well as to measure the combined effects of these XRCC1 variants on glioma risk.

## Introduction

Glioma is the most common and aggressive malignant primary brain tumor in humans, especially in adults, accounting for approximately 30% of all brain and central nervous system (CNS) tumors and 80% of all malignant brain tumors [1], [2]. Currently, the therapy for glioma is a combined approach, using surgery, radiation therapy, and chemotherapy. The prognosis for glioma patients is still poor, except for pilocytic astrocytomas (WHO grade I). Fewer than 3% of glioblastoma patients are still alive at 5 years after diagnosis, with an older age being the most significant and consistent prognostic factor for poorer outcome. Despite decades of research, the etiology of glioma is poorly understood. Many environmental and lifestyle factors including several occupations, environmental carcinogens, and diet have been reported to be associated with an elevated glioma risk, but the only factor unequivocally associated with an increased risk is high dose exposure to ionizing radiation [3], [4]. However, only a minority of those exposed to ionizing radiation eventually develop glioma, suggesting that genetic factors, such as single nucleotide polymorphisms (SNPs), may be crucial to modify the risk for glioma [5], [6].

DNA repair genes play a major role in the DNA mismatch repair pathway, including base excision repair (BER), nucleotide excision repair (NER), mismatch repair (MMR) and double strand break repair (DSBR), and are essential for maintaining the integrity of the genome [7], [8]. The X-ray repair cross-complementing group 1 (XRCC1) gene is an important component of DNA repair and encodes a scaffolding protein that participate in the BER pathway [9]–[11] for repairing small base lesions derived from oxidation and alkylation damage [12]. Several nonsynonymous coding polymorphisms were identified in this gene, and the three which are most extensively studied are Arg399Gln on exon 10 (rs25487, G/A), Arg194Trp on exon 6 (rs1799782, C/T) and Arg280His on exon 9 (rs25489, G/A) [13]. These polymorphisms, which involve amino acid changes at evolutionarily conserved sequences, could alter the function of XRCC1, which may diminish repair kinetics in individuals with the variant alleles and increase the risk of glioma in humans.

To date, several epidemiologic studies have been performed to elucidate the effect of these SNPs on glioma risk. However, the results are to some extent divergent, but nevertheless intriguing. The inconsistency of these studies may be explained by differences in population background, source of controls, sample size, and also by chance. Actually differences in the allele frequencies of these three polymorphisms in Asians and Caucasians have been reported [14], [15]. Since most of the previous association studies focused on Caucasians [16]–[25], few, if any, large-scale studies have been performed in Chinese populations. The genetic effect of XRCC1 polymorphisms on glioma risk in Chinese populations remains largely inconclusive. In addition, several new related studies of glimoa risk in Chinese populations [26]–[28] have since been published. Therefore, in the present study, we performed a meta-analysis to elucidate the relationship between XRCC1 polymorphisms and glioma risk in Chinese populations by combining all available studies.

## Materials and Methods

### Search strategy

We performed a comprehensive literature search of PubMed and EMBASE for relevant studies that tested the association between XRCC1 polymorphisms and the risk of glioma up to August 21, 2014. The following search terms and keywords were used: (“DNA repair gene” OR XRCC1 OR “X-ray repair cross-complementation group 1”) AND (polymorphism OR variant OR variation OR mutation) AND (glioma OR “brain tumor”). In addition, references cited in the retrieved articles were reviewed to trace additional relevant studies missed by the search.

### Inclusion criteria

Included studies were considered eligible if they met all of the following criteria: 1) studies with full text articles; 2) a case–control study evaluating at least one of these three polymorphisms in the XRCC1 gene; 3) enough data to estimate an odds ratio (OR) with 95% confidence interval (CI); 4) no overlapping data. For the studies with the same or overlapping data by the same authors, we selected the ones with the most subjects.

### Data extraction

Data were extracted independently by three investigators. For conflicting evaluations, an agreement was reached following discussion. For each study, the following characteristics were collected: first author, publication year, source of controls, genotyping method, numbers of cases and controls, genotype frequency of cases and controls, and the results of the Hardy–Weinberg equilibrium test.

### Quality score evaluation

The quality of the included studies was independently assessed by three investigators (LWH, RS and LJ) according to the quality assessment criteria (shown in Table S1) that was amended from previous published meta-analyses [29], [30]. All disagreements were resolved by consensus after discussion. Study quality was evaluated on a numerical score ranging from 0 to 12. If the score was ≥7, the study was categorized as “high quality”; otherwise, the study was categorized as “low quality”.

### Statistical analysis

We assessed the deviation from HWE for the genotype distribution in controls using a chi-squared goodness-of-fit test (*P*<0.05 was considered significant). ORs with the corresponding 95% CI were used as the common measures of assessing the strength of association between XRCC1 polymorphisms (Arg399Gln, Arg194Trp, and Arg280His) and glioma risk for each study. The pooled ORs were calculated in an additive model (a allele versus A allele, a was for the minor allele and A was for the major allele), a dominant model (aa+Aa versus AA), recessive model (aa versus Aa+AA) and a codominant model (aa versus AA, Aa versus AA). If the overall gene effect was statistically significant, further comparisons of OR_1_ (aa versus AA), OR_2_ (Aa versus AA) and OR_3_ (aa versus Aa) were explored with a designated as the risk allele. The above pairwise differences were used to determine the most appropriate genetic model. If OR_1_ = OR_3_≠1 and OR_2_ = 1, then a recessive model was indicated. If OR_1_ = OR_2_≠1 and OR_3_ = 1, then a dominant model was indicated. If OR_2_ = 1/OR_3_≠1 and OR_1_ = 1, then a complete over-dominant model was indicated. If OR_1_>OR_2_> 1 and OR_1_>OR_3_>1, or OR_1_<OR_2_<1 and OR_1_<OR_3_<1, then a co-dominant model was indicated [31]. The significance of the pooled ORs was determined using a Z-test, and the level of statistical significance was established as *P*<0.05. The heterogeneity among studies was checked by the Q test [32]. The *I^2^* statistic, which is a quantitative measure of the proportion of the total variation across studies due to heterogeneity [33], was also calculated. If the *P* value for the heterogeneity test was greater than 0.05, the Mantel–Haenszel method-based fixed effects model [34] was used to calculate the pooled OR. Otherwise, the DerSimonian and Laird method-based random effects model [35] was performed. Sensitivity analysis was performed by limiting the meta-analysis to studies conforming to HWE and omitting each study in turn to assess the stability of results, respectively. Potential publication bias was evaluated by visual inspection of the Begg funnel plots in which the standard error of log (OR) of each study was plotted against its log (OR). We also performed an Egger's linear regression test (*P*<0.05 was considered a significant publication bias) [36]. All of the statistical analyses were performed using a software program, STATA version 11.0 (Stata, College Station, TX, USA).

## Results

### Extraction process and study characteristics

**Figure 1:**
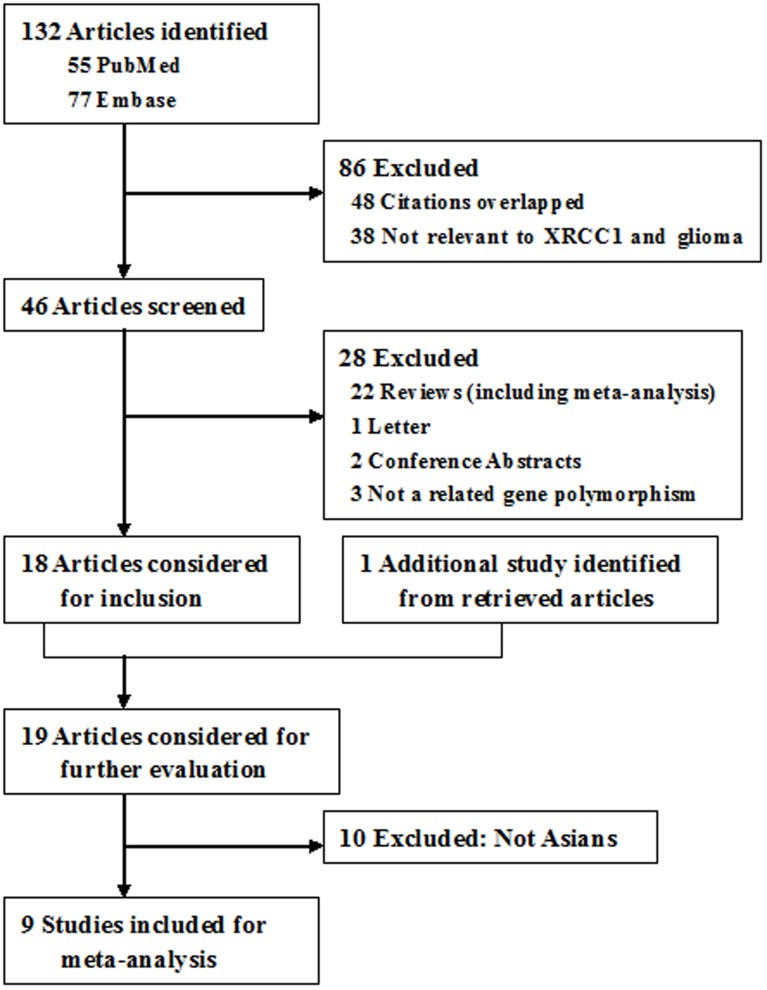
Flow of Included Studies.

According to our search criterion, 132 articles were retrieved. Among them, the majority were excluded after the first screening based on abstracts or titles, mainly because they were overlapped citations, not relevant to the XRCC1 polymorphisms and glioma risk, reviews, conference abstracts, or not a related gene polymorphism. Afterwards, a total of 19 full-text articles [16]–[28], [37]–[42] were preliminarily identified for further detailed evaluation (Figure 1). Of these, 10 studies were excluded [16]–[25] because the country of source was not from China. Eventually, nine case-control studies [26]–[28], [37]–[42] were selected, including 8 studies for the Arg399Gln polymorphism (3062 cases and 3362 controls), 8 studies for the Arg194Trp polymorphism (3419 cases and 3680 controls), and 5 studies for the Arg280His polymorphism (2234 cases and 2380 controls). With respect to the assessment of study quality, the vast majority of the included studies were high quality (shown in Table S2) except for the study by Liu *et al.* [41]. The characteristics of these included studies and the genotype distribution and allele frequency of XRCC1 polymorphisms in case and control subjects is shown in Table 1.

**Table 1:** Characteristics of studies included in the meta-analysis and their genotype distributions of XRCC1 polymorphisms.

Polymorphism	First author	Year	Design	Sample size (case/control)		Case			Control			HWE in control	MAF
						AA	Aa	aa	AA	Aa	aa		
**Arg399Gln**	Gao	2014	HB	326	375	126	155	45	178	168	29	0.215	0.301
	Xu	2013	HB	886	886	451	365	70	469	372	45	0.008	0.261
	Pan	2013	HB	443	443	226	190	27	244	178	21	0.108	0.248
	Luo	2013	HB	296	415	111	134	51	189	181	45	0.866	0.327
	Wang	2012	HB	624	580	270	279	75	300	232	48	0.739	0.283
	Zhou	2011	HB	271	289	121	113	37	147	118	24	0.963	0.287
	Hu	2011	HB	127	249	58	48	21	145	75	29	<0.001	0.267
	Liu	2011	HB	89	89	23	37	29	28	34	27	0.026	0.494
**Arg194Trp**	Gao	2014	HB	326	376	235	73	18	279	84	13	0.041	0.146
	Xu	2013	HB	886	886	525	301	60	540	311	35	0.236	0.215
	Pan	2013	HB	444	443	301	116	27	327	101	15	0.045	0.148
	Luo	2013	HB	297	415	204	63	30	297	96	22	<0.001	0.169
	Liu	2012	HB	444	442	294	105	45	334	89	19	<0.001	0.144
	Wang	2012	HB	624	580	376	218	30	355	205	20	0.143	0.211
	Zhou	2011	HB	271	289	145	112	14	159	117	13	0.138	0.247
	Hu	2011	HB	127	249	71	38	18	163	64	22	<0.001	0.217
**Arg280His**	Gao	2014	HB	326	376	250	66	10	313	57	6	0.079	0.092
	Xu	2013	HB	886	886	618	177	91	621	178	87	<0.001	0.199
	Wang	2012	HB	624	580	506	115	3	473	98	9	0.140	0.100
	Zhou	2011	HB	271	289	218	45	8	240	44	5	0.085	0.093
	Hu	2011	HB	127	249	72	28	27	153	58	38	<0.001	0.269

Abbreviations: HWE, Hardy-Weinberg equilibrium; HB, hospital-based; MAF, minor allele frequency; A, the major allele; a, the minor allele.

### Meta-analysis results

**Figure 2:**
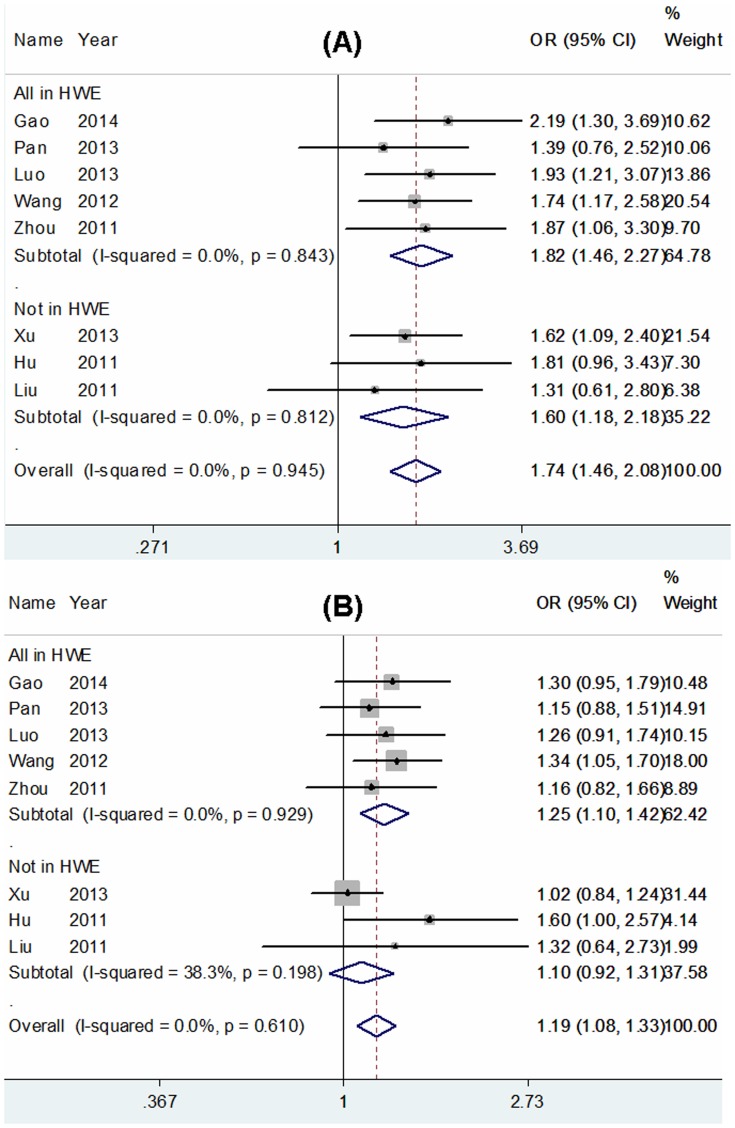
Forest plots of ORs with 95% CI for XRCC1 Arg399Gln polymorphism and the risk of glioma observed in Chinese population (fixed effects). The center of each square represents the OR, the area of the square is the number of sample and thus the weight used in the meta-analysis, and the horizontal line indicates the 95%CI. (A) Gln/Gln vs. Arg/Arg. (B) Arg/Gln vs. Arg/Arg.

**Figure 3:**
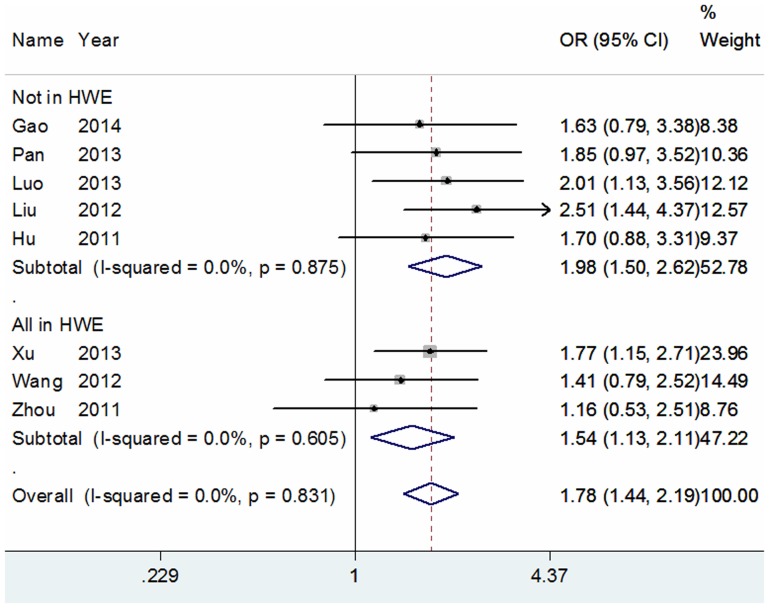
Forest plots of ORs with 95% CI for XRCC1 Arg194Trp polymorphism and the risk of glioma observed in recessive model among Chinese (fixed effects). The center of each square represents the OR, the area of the square is the number of sample and thus the weight used in the meta-analysis, and the horizontal line indicates the 95%CI.

The main results of the meta-analysis are shown in Table 2. According to the principle of genetic model selection by Thakkinstian *et al.* [31], the most appropriate genetic model for the Arg399Gln/Arg194Trp polymorphisms was the codominant model and the recessive model, respectively. Our results revealed that the Arg399Gln polymorphism was significantly associated with an increased risk of glioma in the Chinese population (Gln/Gln vs. Arg/Arg: OR = 1.82, 95% CI = 1.46–2.27, *P* = 0.000; Arg/Gln vs. Arg/Arg: OR = 1.25, 95% CI = 1.10–1.42, *P* = 0.001; recessive model: OR = 1.63, 95% CI = 1.32–2.01, *P* = 0.000; dominant model: OR = 1.34, 95% CI = 1.18–1.51, *P* = 0.000; additive model: OR = 1.31, 95% CI = 1.19–1.44, *P* = 0.000; Figure 2, Table 2). For the Arg194Trp polymorphism, a significant association between this polymorphism and glioma risk was also observed (Trp/Trp vs. Arg/Arg: OR = 1.82, 95% CI = 1.48–2.25, *P* = 0.000; recessive model: OR = 1.78, 95% CI = 1.44–2.19, *P* = 0.000; dominant model: OR = 1.17, 95% CI = 1.06–1.30, *P* = 0.001; additive model: OR = 1.23, 95% CI = 1.13–1.33, *P* = 0.000; Figure 3, Table 2), with the exception of the heterozygote comparison model (OR = 1.08, 95% CI = 0.97–1.20, *P* = 0.169, Table 2). But, for the Arg280His polymorphism, we did not detect any significant association with glioma risk in any genetic model (Table 2). Since several original papers depart from the HWE which could cause unreliable results, we performed stratification analysis according to the status of HWE. Because ethnicity of all studies was Chinese and the source of controls was hospital-based, we did not carry out subgroup analysis. In addition, the subgroup analysis according to quality assessment scores is not shown because only one included study was low quality which did not materially change the corresponding pooled ORs.

**Table 2:** Results of meta-analysis for Arg399Gln, Arg194Trp and Arg280His polymorphisms and the risk of glioma in Chinese population.

Genetic model		Recessive model				Dominant model				Homozygote				Heterozygote				Additive model			
**Arg399Gln**	n	Gln/Gln vs. Arg/Gln + Arg/Arg				Gln/Gln + Arg/Gln vs. Arg/Arg				Gln/Gln vs. Arg/Arg				Arg/Gln vs. Arg/Arg				Gln vs. Arg			
		OR(95%CI)	*P*_OR_	*I* ^2^(%)	*P*_h_	OR(95%CI)	*P*_OR_	*I* ^2^(%)	*P*_h_	OR(95%CI)	*P*_OR_	*I* ^2^(%)	*P*_h_	OR(95%CI)	*P*_OR_	*I* ^2^(%)	*P*_h_	OR(95%CI)	*P*_OR_	*I* ^2^(%)	*P*_h_
Total	8(3062/3326)	1.57(1.32–1.86)	0.000	0.0	0.926	1.27(1.15–1.41)	0.000	0.0	0.506	1.74(1.46–2.08)	0.000	0.0	0.945	1.19(1.08–1.33)	0.001	0.0	0.610	1.26(1.17–1.36)	0.000	0.0	0.567
All in HWE	5(1960/2102)	1.63(1.32–2.01)	0.000	0.0	0.877	1.34(1.18–1.51)	0.000	0.0	0.842	1.82(1.46–2.27)	0.000	0.0	0.843	1.25(1.10–1.42)	0.001	0.0	0.929	1.31(1.19–1.44)	0.000	0.0	0.750
Not in HWE	3(1102/1224)	1.46(1.10–1.96)	0.010	0.0	0.621	1.17(0.99–1.38)	0.063	39.1	0.194	1.60(1.18–2.18)	0.003	0.0	0.812	1.10(0.92–1.31)	0.281	38.3	0.198	1.18(1.04–1.35)	0.010	16.2	0.303
**Arg194Trp**	n	Trp/Trp vs. Arg/Trp + Arg/Arg				Trp/Trp + Arg/Trp vs. Arg/Arg				Trp/Trp vs. Arg/Arg				Arg/Trp vs. Arg/Arg				Trp vs. Arg			
		OR(95%CI)	*P*_OR_	*I* ^2^(%)	*P*_h_	OR(95%CI)	*P*_OR_	*I* ^2^(%)	*P*_h_	OR(95%CI)	*P*_OR_	*I* ^2^(%)	*P*_h_	OR(95%CI)	*P*_OR_	*I* ^2^(%)	*P*_h_	OR(95%CI)	*P*_OR_	*I* ^2^(%)	*P*_h_
Total	8(3419/3680)	1.78(1.44–2.19)	0.000	0.0	0.831	1.17(1.06–1.30)	0.001	15.0	0.312	1.82(1.48–2.25)	0.000	0.0	0.782	1.08(0.97–1.20)	0.169	0.0	0.666	1.23(1.13–1.33)	0.000	42.7	0.094
All in HWE	3(1781/1755)	1.54(1.13–2.11)	0.006	0.0	0.605	1.06(0.93–1.21)	0.392	0.0	0.980	1.55(1.13–2.13)	0.007	0.0	0.641	1.01(0.88–1.16)	0.920	0.0	0.966	1.10(0.99–1.23)	0.089	0.0	0.850
Not in HWE	5(1638/1925)	1.98(1.50–2.62)	0.000	0.0	0.875	1.32(1.14–1.53)	0.000	0.0	0.485	2.07(1.56–2.75)	0.000	0.0	0.852	1.17(1.00–1.38)	0.049	0.0	0.578	1.40(1.24–1.58)	0.000	1.5	0.398
**Arg280His**	n	His/His vs. Arg/His + Arg/Arg				His/His + Arg/His vs. Arg/Arg				His/His vs. Arg/Arg				Arg/His vs. Arg/Arg				His vs. Arg			
		OR(95%CI)	*P*_OR_	*I* ^2^(%)	*P*_h_	OR(95%CI)	*P*_OR_	*I* ^2^(%)	*P*_h_	OR(95%CI)	*P*_OR_	*I* ^2^(%)	*P*_h_	OR(95%CI)	*P*_OR_	*I* ^2^(%)	*P*_h_	OR(95%CI)	*P*_OR_	*I* ^2^(%)	*P*_h_
Total	5(2234/2380)	1.14(0.89–1.46)	0.306	39.9	0.155	1.11(0.97–1.27)	0.128	0.0	0.424	1.14(0.89–1.47)	0.295	41.6	0.144	1.10(0.94–1.28)	0.224	0.0	0.623	1.30(1.00–1.69)	0.053	77.1	0.002
All in HWE	3(1221/1245)	1.11(0.60–2.05)	0.740	63.3	0.065	1.19(0.97–1.46)	0.090	20.3	0.285	1.15(0.62–2.12)	0.658	64.6	0.059	1.19(0.97–1.47)	0.096	0.0	0.517	1.46(0.90–2.36)	0.124	84.3	0.002
Not in HWE	2(1013/1135)	1.14(0.87–1.50)	0.331	18.2	0.269	1.05(0.87–1.26)	0.606	0.0	0.460	1.14(0.87–1.50)	0.343	16.6	0.274	1.00(0.81–1.25)	0.974	0.0	0.929	1.07(0.93–1.24)	0.346	35.6	0.213

*P*_OR_ *P* values for pooled OR from Z-test. *P*_h_ *P* values for heterogeneity from *Q* test. *I^2^*, the percentage of variability in OR attributable to heterogeneity. Random-effects model was used when *P* value for heterogeneity test <0.05; otherwise, fixed-model was used.

### Test of heterogeneity and sensitivity analyses

The results of heterogeneity test indicated that there was no significant heterogeneity for the Arg399Gln/Arg194Trp polymorphisms across studies. However, we found heterogeneity for the Arg280His polymorphism only in an additive model (*P*_h_ = 0.002, *I^2^* = 77.1%). To explore the potential sources of heterogeneity across studies, we determined that the study by Zhou *et al.* [40] could contribute to substantial heterogeneity because heterogeneity was significantly decreased, in the additive model (*P*_h_ = 0.117, *I^2^* = 49.0%), after exclusion of this study. Although there were 3 and 2 studies that deviated from HWE for the Arg399Gln/Arg280His polymorphisms, respectively, the corresponding pooled ORs were not materially altered by including or not including these studies (Table 2). Similarly, the results of the Arg194Trp polymorphism remained practically unchanged in a recessive model and a codominant model when excluding the 5 studies that departed from HWE. Nevertheless, this polymorphism was no longer associated with the risk of glioma in a dominant model (OR = 1.06, 95% CI = 0.93–1.21, *P* = 0.392) and an additive model (OR = 1.10, 95% CI = 0.99–1.23, *P* = 0.089). Additionally, we also assessed the influence of each individual study on the pooled ORs by sequential omission of individual studies. The results showed the pooled ORs of these three polymorphisms were not materially altered by the contribution of any individual study, suggesting that the results of this meta-analysis are credible (data also not shown).

### Publication bias

**Figure 4:**
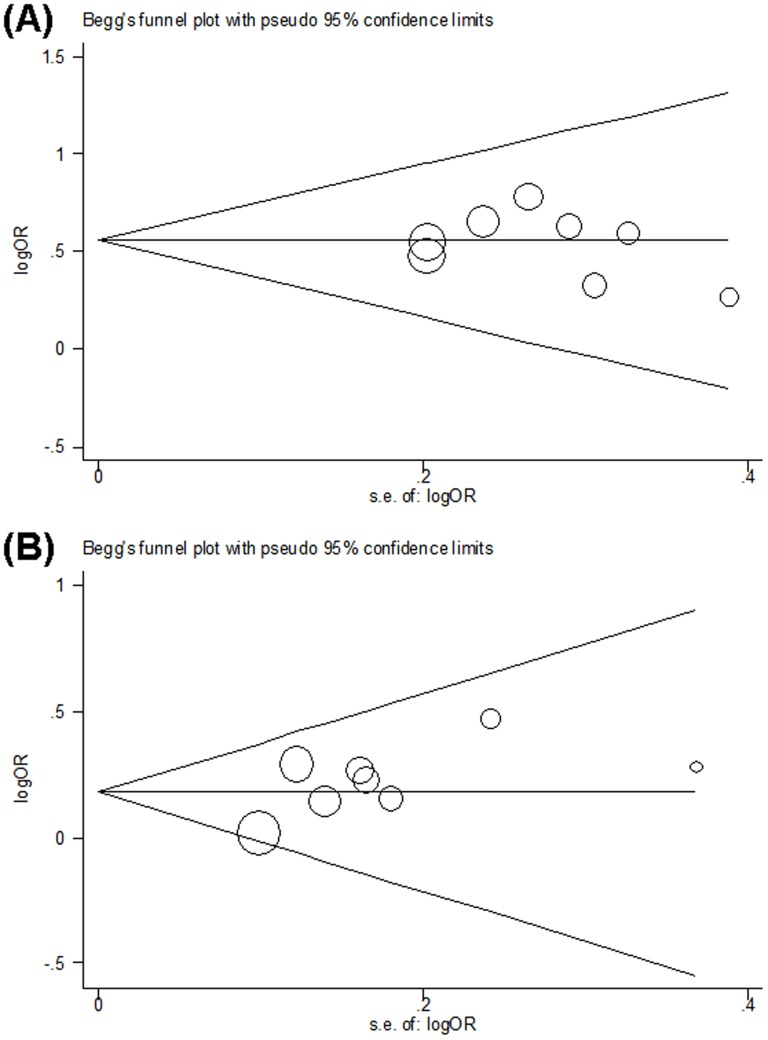
Begg's funnel plots of Arg399Gln polymorphism and glioma risk for publication bias test. Each point represents a separate study for the indicated association. Log (OR), natural logarithm of OR. Horizontal line, mean effect size. (A) Gln/Gln vs. Arg/Arg. (B) Arg/Gln vs. Arg/Arg.

Publication bias was assessed by performing Funnel plot and Egger's regression tests under all contrast models. All of these three genetic polymorphisms showed consistent results, indicating no publication bias. Usinge the Arg399Gln polymorphism as an example; the shapes of the funnel plot did not indicate any evidence of obvious asymmetry in a codominant model (Figure 4), and the Egger's test also suggested that there was no evidence of publication bias (*P* = 0.185 for a dominant model, *P* = 0.296 for a recessive model, *P* = 0.300, or for an additive model, *P* = 0.108 for Arg/Gln vs. Arg/Arg and *P* = 0.552 for Gln/Gln vs. Arg/Arg, respectively).

## Discussion

DNA damage, which leads to gene deletions, amplifications, rearrangements, and translocations occurs very frequently and results in the formation of a tumor [7], [43]. Many of these mutations may lead to less effective DNA repair than normal. It is acknowledged that glioma is appreciably associated with specific mutations causing by exposure to ionizing radiation in the DNA mismatch repair pathway. XRCC1 is an essential DNA repair gene involved in BER pathway and the vast majority of previous studies have been focused on three polymorphisms (Arg399Gln, Arg194Trp, and Arg280His) in this gene. Genetic variations in this gene confers a susceptibility to tumorogeneis through the alteration of base excision repair functions [44]. At present, several systematic reviews and meta-analyses have been carried out as preliminary studies to determine the association between XRCC1 variants and glioma risk based on pervious published studies [45]–[54]. However, none of these studies collected sufficient data to draw a solid conclusion in a Chinese population and some results remain contradictory. Thus, Zhang et al. [52] reported that XRCC1 Arg194Trp polymorphism was not a risk factor for glioma risk in a Chinese population, which was the opposite of the conclusions made in a previous study [51]. Considering the paradoxical and underpowered conclusions of the individual studies, we conducted the most comprehensive meta-analysis using available eligible data to provide more reliable results to determine the association between the variants of the XRCC1 gene and glioma risk in the Chinese population.

Overall, our combined results based on available data from of all the studies revealed that the Arg399Gln polymorphism in XRCC1 gene was associated with increased risk of glioma among Chinese people in all genetic models, which was consistent with the conclusion of individual studies involving the Arg399Gln polymorphism [26]–[28], [37]–[41]. Meanwhile, we also detected that individuals harboring the Trp/Trp genotype of the Arg194Trp polymorphism might have an increased risk of developing glioma, which was in line with the majority, but not all, previous studies [26]–[28], [37], [38], [51]. As for the Arg280His polymorphism, our results did not provide any evidence of such an association with glioma risk in any genetic model, which coincided with the conclusions of all previous studies [28], [37], [39], [40]. For example, Xu *et al.* [28] suggested that the Arg280His polymorphism was unlikely to be associated with the risk of glioma.

It is generally agreed that departures from HWE in controls may be due to genotyping error, chance, nonrandom mating, genetic drifting, population stratification, and selection bias. Although there were 3, 2 and 5 studies that deviated from HWE for the Arg399Gln, Arg280His, and Arg194Trp polymorphisms, respectively, the studies that appeared to deviate from HWE should not be excluded mechanically in the meta-analysis unless there are other convincing grounds for doubting the quality of the study [55]. Also, there is no consensus on what to do with studies that are not in HWE in the meta-analysis of genetic association studies. Some authors suggest performing sensitivity analyses, pooling both with and without the studies that appear not to be in HWE and assessing whether studies classified as not being in HWE provide a different estimate of the genetic effect [56], [57]. Furthermore, Mao *et al.* [58] emphasized that authors of gene-disease association meta-analyses may need to pay more attention to HWE issues, and sensitivity analyses including and excluding the HWE-violating studies may need to be routinely performed in meta-analyses of genetic association studies. In this study we performed sensitivity analyses by excluding the HWE-violating studies to check the robustness of our conclusions, and the corresponding pooled ORs were not materially altered. In addition, we comprehensively assessed the publication bias using several means including the Begg's and Egger's tests as well as funnel plot tests, indicating no publication bias for all these three genetic polymorphisms. In view of this, we are strongly convinced that the methods are appropriate and well described and the results or data of our meta-analysis, in essence, are sound and reliable.

Additionally, there is still a lack of uniform and standardized quality score methods for evaluating case-control gene association studies although it is crucial for a meta-analysis to assess the quality of the individual included studies. Here we used a self-made rating scale for study quality assessment, which was modified based on two previously published meta-analyses [29], [30]. The quality score assessment results showed that almost all of individual studies were high quality except for the study by Liu *et al.* [41], indicating that the quality of the included studies was generally high, which lends support to our conclusions. However, considering that high-quality studies may offer quite different outcomes from that of low-quality studies [59], we recommend that researchers carry out study quality assessment and stratification analysis based on the quality appraisal scores when performing the quantitative synthesis of the genetic polymorphism association studies.

When interpreting the results of the current study, some limitations should be addressed. First, lacking the original data for the included studies limited our further evaluation of the association between glioma risk and other risk factors, such as age, gender, smoking status, alcohol consumption and other variables, which might have caused a serious confounding bias. Second, we did not estimate the potential interactions among gene–gene, gene–environment, or even between various polymorphic loci of the same gene, which may alter the risk of cancer. Although the analysis of haplotypes can increase the power to detect disease associations, our study was limited to analyzing a single SNP site owing to only one study [37] focused on determining the XRCC1 haplotype. Third, selection bias should be considered because the controls from the primary literatures were all hospital-based which may not be very representative of the general population. Finally, some inevitable publication bias might exist in the results because only published studies were retrieved although the funnel plot and Egger's test indicated no remarkable publication bias.

In summary, this meta-analysis provides evidence that both the Arg399Gln and Arg194Trp polymorphisms may contribute to genetic susceptibility to glioma risk in the Chinese population, whereas Arg280His polymorphism may have no impact. Nevertheless, large-scale, well-designed and population-based studies are needed to investigate the combined effects of these variants within XRCC1 gene or other BER genes in the Chinese population, which may eventually lead to better comprehensive understanding of their possible roles in gliomagenesis.

## Supporting Information

Table S1 **Scale for Quality Assessment.**
(DOC)

Table S2 **Quality score assessment results.**
(DOC)

Checklist S1 **Prisma 2009 Checklist for this meta-analysis.**
(DOC)

## References

[1] Goodenberger ML, Jenkins RB (2012) Genetics of adult glioma. Cancer genetics 205: 613–621. 23238284 10.1016/j.cancergen.2012.10.009

[2] Ricard D, Idbaih A, Ducray F, Lahutte M, Hoang-Xuan K, et al (2012) Primary brain tumours in adults. Lancet 379: 1984–1996. 22510398 10.1016/S0140-6736(11)61346-9

[3] Ostrom QT, Barnholtz-Sloan JS (2011) Current state of our knowledge on brain tumor epidemiology. Current neurology and neuroscience reports 11: 329–335. 21336822 10.1007/s11910-011-0189-8

[4] Schwartzbaum JA, Fisher JL, Aldape KD, Wrensch M (2006) Epidemiology and molecular pathology of glioma. Nature clinical practice Neurology 2: : 494–503; quiz 491 p following 516. 10.1038/ncpneuro0289 16932614

[5] Gu J, Liu Y, Kyritsis AP, Bondy ML (2009) Molecular epidemiology of primary brain tumors. Neurotherapeutics: the journal of the American Society for Experimental NeuroTherapeutics 6: 427–435. 19560733 10.1016/j.nurt.2009.05.001 PMC5084179

[6] Shete S, Hosking FJ, Robertson LB, Dobbins SE, Sanson M, et al (2009) Genome-wide association study identifies five susceptibility loci for glioma. Nature genetics 41: 899–904. 19578367 10.1038/ng.407 PMC4501476

[7] Wood RD, Mitchell M, Sgouros J, Lindahl T (2001) Human DNA repair genes. Science (New York, NY) 291: 1284–1289. 10.1126/science.1056154 11181991

[8] Yu Z, Chen J, Ford BN, Brackley ME, Glickman BW (1999) Human DNA repair systems: an overview. Environmental and molecular mutagenesis 33: 3–20. 10037319 10.1002/(sici)1098-2280(1999)33:1<3::aid-em2>3.0.co;2-l

[9] Caldecott KW, Tucker JD, Stanker LH, Thompson LH (1995) Characterization of the XRCC1-DNA ligase III complex in vitro and its absence from mutant hamster cells. Nucleic acids research 23: 4836–4843. 8532526 10.1093/nar/23.23.4836 PMC307472

[10] Campalans A, Marsin S, Nakabeppu Y, O'Connor T R, Boiteux S, et al (2005) XRCC1 interactions with multiple DNA glycosylases: a model for its recruitment to base excision repair. DNA repair 4: 826–835. 15927541 10.1016/j.dnarep.2005.04.014

[11] Siciliano MJ, Carrano AV, Thompson LH (1986) Assignment of a human DNA-repair gene associated with sister-chromatid exchange to chromosome 19. Mutation research 174: 303–308. 3736579 10.1016/0165-7992(86)90051-5

[12] Almeida KH, Sobol RW (2007) A unified view of base excision repair: lesion-dependent protein complexes regulated by post-translational modification. DNA repair 6: 695–711. 17337257 10.1016/j.dnarep.2007.01.009 PMC1995033

[13] Shen MR, Jones IM, Mohrenweiser H (1998) Nonconservative amino acid substitution variants exist at polymorphic frequency in DNA repair genes in healthy humans. Cancer research 58: 604–608. 9485007

[14] Hamajima N, Takezaki T, Tajima K (2002) Allele Frequencies of 25 Polymorphisms Pertaining to Cancer Risk for Japanese, Koreans and Chinese. Asian Pacific journal of cancer prevention: APJCP 3: 197–206. 12718576

[15] Moullan N, Cox DG, Angele S, Romestaing P, Gerard JP, et al (2003) Polymorphisms in the DNA repair gene XRCC1, breast cancer risk, and response to radiotherapy. Cancer epidemiology, biomarkers & prevention: a publication of the American Association for Cancer Research, cosponsored by the American Society of Preventive Oncology 12: 1168–1174. 14652276

[16] Bethke L, Webb E, Murray A, Schoemaker M, Johansen C, et al (2008) Comprehensive analysis of the role of DNA repair gene polymorphisms on risk of glioma. Human molecular genetics 17: 800–805. 18048407 10.1093/hmg/ddm351

[17] Cengiz SL, Acar H, Inan Z, Yavuz S, Baysefer A (2008) Deoxy-ribonucleic acid repair genes XRCC1 and XPD polymorphisms and brain tumor risk. Neurosciences (Riyadh, Saudi Arabia) 13: 227–232. 21063329

[18] Felini MJ, Olshan AF, Schroeder JC, North KE, Carozza SE, et al (2007) DNA repair polymorphisms XRCC1 and MGMT and risk of adult gliomas. Neuroepidemiology 29: 55–58. 17898525 10.1159/000108919

[19] Karahalil B, Bohr VA, Wilson DM 3rd (2012) Impact of DNA polymorphisms in key DNA base excision repair proteins on cancer risk. Human & experimental toxicology 31: 981–1005. 23023028 10.1177/0960327112444476 PMC4586256

[20] Kiuru A, Lindholm C, Heinavaara S, Ilus T, Jokinen P, et al (2008) XRCC1 and XRCC3 variants and risk of glioma and meningioma. Journal of neuro-oncology 88: 135–142. 18330515 10.1007/s11060-008-9556-y

[21] Liu Y, Scheurer ME, El-Zein R, Cao Y, Do KA, et al (2009) Association and interactions between DNA repair gene polymorphisms and adult glioma. Cancer epidemiology, biomarkers & prevention: a publication of the American Association for Cancer Research, cosponsored by the American Society of Preventive Oncology 18: 204–214. 10.1158/1055-9965.EPI-08-0632 PMC2917049 19124499

[22] Rajaraman P, Hutchinson A, Wichner S, Black PM, Fine HA, et al (2010) DNA repair gene polymorphisms and risk of adult meningioma, glioma, and acoustic neuroma. Neuro-oncology 12: 37–48. 20150366 10.1093/neuonc/nop012 PMC2940551

[23] Rodriguez-Hernandez I, Perdomo S, Santos-Briz A, Garcia JL, Gomez-Moreta JA, et al.. (2013) Analysis of DNA repair gene polymorphisms in glioblastoma. Gene. 10.1016/j.gene.2013.11.077 24325908

[24] Wang LE, Bondy ML, Shen H, El-Zein R, Aldape K, et al (2004) Polymorphisms of DNA repair genes and risk of glioma. Cancer research 64: 5560–5563. 15313891 10.1158/0008-5472.CAN-03-2181

[25] Yosunkaya E, Kucukyuruk B, Onaran I, Gurel CB, Uzan M, et al (2010) Glioma risk associates with polymorphisms of DNA repair genes, XRCC1 and PARP1. British journal of neurosurgery 24: 561–565. 20868244 10.3109/02688697.2010.489655

[26] Luo KQ, Mu SQ, Wu ZX, Shi YN, Peng JC (2013) Polymorphisms in DNA repair genes and risk of glioma and meningioma. Asian Pacific journal of cancer prevention: APJCP 14: 449–452. 23534771 10.7314/apjcp.2013.14.1.449

[27] Pan WR, Li G, Guan JH (2013) Polymorphisms in DNA repair genes and susceptibility to glioma in a chinese population. International journal of molecular sciences 14: 3314–3324. 23385236 10.3390/ijms14023314 PMC3588045

[28] Xu G, Wang M, Xie W, Bai X (2013) Three polymorphisms of DNA repair gene XRCC1 and the risk of glioma: a case-control study in northwest China. Tumour biology: the journal of the International Society for Oncodevelopmental Biology and Medicine. 10.1007/s13277-013-1191-3 24048757

[29] Gao LB, Pan XM, Li LJ, Liang WB, Bai P, et al (2011) Null genotypes of GSTM1 and GSTT1 contribute to risk of cervical neoplasia: an evidence-based meta-analysis. PloS one 6: e20157. 21629772 10.1371/journal.pone.0020157 PMC3100325

[30] Yang X, Long S, Deng J, Deng T, Gong Z, et al (2013) Glutathione S-transferase polymorphisms (GSTM1, GSTT1 and GSTP1) and their susceptibility to renal cell carcinoma: an evidence-based meta-analysis. PloS one 8: e63827. 23717494 10.1371/journal.pone.0063827 PMC3661732

[31] Thakkinstian A, McElduff P, D'Este C, Duffy D, Attia J (2005) A method for meta-analysis of molecular association studies. Stat Med 24: 1291–1306. 15568190 10.1002/sim.2010

[32] Cochran W (1954) The combination of estimates from different experiments. Biometrics 10: 101–129.

[33] Higgins JP, Thompson SG, Deeks JJ, Altman DG (2003) Measuring inconsistency in meta-analyses. BMJ (Clinical research ed) 327: 557–560. 10.1136/bmj.327.7414.557 PMC192859 12958120

[34] Mantel N, Haenszel W (1959) Statistical aspects of the analysis of data from retrospective studies of disease. Journal of the National Cancer Institute 22: 719–748. 13655060

[35] DerSimonian R, Laird N (1986) Meta-analysis in clinical trials. Controlled clinical trials 7: 177–188. 3802833 10.1016/0197-2456(86)90046-2

[36] Egger M, Davey Smith G, Schneider M, Minder C (1997) Bias in meta-analysis detected by a simple, graphical test. BMJ (Clinical research ed) 315: 629–634. 10.1136/bmj.315.7109.629 PMC2127453 9310563

[37] Hu XB, Feng Z, Fan YC, Xiong ZY, Huang QW (2011) Polymorphisms in DNA repair gene XRCC1 and increased genetic susceptibility to glioma. Asian Pacific journal of cancer prevention: APJCP 12: 2981–2984. 22393975

[38] Liu HB, Peng YP, Dou CW, Su XL, Gao NK, et al (2012) Comprehensive study on associations between nine SNPs and glioma risk. Asian Pacific journal of cancer prevention: APJCP 13: 4905–4908. 23244079 10.7314/apjcp.2012.13.10.4905

[39] Wang D, Hu Y, Gong H, Li J, Ren Y, et al (2012) Genetic polymorphisms in the DNA repair gene XRCC1 and susceptibility to glioma in a Han population in northeastern China: a case-control study. Gene 509: 223–227. 22951806 10.1016/j.gene.2012.08.023

[40] Zhou LQ, Ma Z, Shi XF, Yin XL, Huang KX, et al (2011) Polymorphisms of DNA repair gene XRCC1 and risk of glioma: a case-control study in Southern China. Asian Pacific journal of cancer prevention: APJCP 12: 2547–2550. 22320953

[41] Liu J, Sun H, Huang L, Hu P, Dai X (2011) Relationship between XRRC1 polymorphisms and adult gliomas. Mod Pre Med 38: 3340–3341.

[42] Gao K, Mu SQ, Wu ZX (2014) Investigation of the effects of single-nucleotide polymorphisms in DNA repair genes on the risk of glioma. Genet Mol Res 13: 1203–1211. 24634177 10.4238/2014.February.27.5

[43] De Bont R, van Larebeke N (2004) Endogenous DNA damage in humans: a review of quantitative data. Mutagenesis 19: 169–185. 15123782 10.1093/mutage/geh025

[44] Monaco R, Rosal R, Dolan MA, Pincus MR, Brandt-Rauf PW (2007) Conformational effects of a common codon 399 polymorphism on the BRCT1 domain of the XRCC1 protein. The protein journal 26: 541–546. 17899335 10.1007/s10930-007-9095-y

[45] Jiang L, Fang X, Bao Y, Zhou JY, Shen XY, et al (2013) Association between the XRCC1 polymorphisms and glioma risk: a meta-analysis of case-control studies. PloS one 8: e55597. 23383237 10.1371/journal.pone.0055597 PMC3559473

[46] Li M, Zhou Q, Tu C, Jiang Y (2013) A meta-analysis of an association between the XRCC1 polymorphisms and gliomas risk. Journal of neuro-oncology 111: 221–228. 23238971 10.1007/s11060-012-1022-1

[47] Martinez R (2012) Beyond Genetics in Glioma Pathways: The Ever-Increasing Crosstalk between Epigenomic and Genomic Events. Journal of signal transduction 2012: 519807. 22778947 10.1155/2012/519807 PMC3385669

[48] Sun JY, Zhang CY, Zhang ZJ, Dong YF, Zhang AL, et al (2012) Association between XRCC1 gene polymorphisms and risk of glioma development: a meta-analysis. Asian Pacific journal of cancer prevention: APJCP 13: 4783–4788. 23167420 10.7314/apjcp.2012.13.9.4783

[49] Wei X, Chen D, Lv T (2013) A functional polymorphism in XRCC1 is associated with glioma risk: evidence from a meta-analysis. Molecular biology reports 40: 567–572. 23096083 10.1007/s11033-012-2093-y

[50] Yi L, Xiao-Feng H, Yun-Tao L, Hao L, Ye S, et al (2013) Association between the XRCC1 Arg399Gln Polymorphism and Risk of Cancer: Evidence from 297 Case-Control Studies. PloS one 8: e78071. 24205095 10.1371/journal.pone.0078071 PMC3812151

[51] Zhang H, Liu H, Knauss JL (2013) Associations between three XRCC1 polymorphisms and glioma risk: a meta-analysis. Tumour biology: the journal of the International Society for Oncodevelopmental Biology and Medicine 34: 3003–3013. 23712607 10.1007/s13277-013-0865-1

[52] Zhang L, Wang Y, Qiu Z, Luo J, Zhou Z, et al (2012) The XRCC1 Arg194Trp polymorphism is not a risk factor for glioma: A meta-analysis involving 1,440 cases and 2,562 controls. Experimental and therapeutic medicine 4: 1057–1062. 23226774 10.3892/etm.2012.716 PMC3494116

[53] Zhang L, Wang Y, Qiu Z, Luo J, Zhou Z, et al (2013) XRCC1 Arg280His polymorphism and glioma risk: A meta-analysis involving 1439 cases and 2564 controls. Pakistan journal of medical sciences 29: 37–42. 24353504 10.12669/pjms.291.2694 PMC3809186

[54] Zhu W, Yao J, Li Y, Xu B (2013) Assessment of the association between XRCC1 Arg399Gln polymorphism and glioma susceptibility. Tumour biology: the journal of the International Society for Oncodevelopmental Biology and Medicine. 10.1007/s13277-013-1397-4 24258108

[55] Minelli C, Thompson JR, Abrams KR, Thakkinstian A, Attia J (2008) How should we use information about HWE in the meta-analyses of genetic association studies? Int J Epidemiol 37: 136–146. 18037675 10.1093/ije/dym234

[56] Attia J, Thakkinstian A, D'Este C (2003) Meta-analyses of molecular association studies: methodologic lessons for genetic epidemiology. J Clin Epidemiol 56: 297–303. 12767405 10.1016/s0895-4356(03)00011-8

[57] Salanti G, Amountza G, Ntzani EE, Ioannidis JP (2005) Hardy-Weinberg equilibrium in genetic association studies: an empirical evaluation of reporting, deviations, and power. Eur J Hum Genet 13: 840–848. 15827565 10.1038/sj.ejhg.5201410

[58] Mao C, Liao RY, Chen Q (2010) Sensitivity analyses including and excluding the HWE-violating studies are required for meta-analyses of genetic association studies. Breast Cancer Res Treat 121: 245–246. 19866356 10.1007/s10549-009-0605-9

[59] Camargo MC, Mera R, Correa P, Peek RM Jr, Fontham ET, et al (2006) Interleukin-1beta and interleukin-1 receptor antagonist gene polymorphisms and gastric cancer: a meta-analysis. Cancer epidemiology, biomarkers & prevention: a publication of the American Association for Cancer Research, cosponsored by the American Society of Preventive Oncology 15: 1674–1687. 10.1158/1055-9965.EPI-06-0189 16985030

