# Supplementary material for: XRCC1 Gene Polymorphisms and Glioma Risk in Chinese Population: A Meta-Analysis
Source: PLoS One. 2014 Nov 6;9(11):e111981. doi: 10.1371/journal.pone.0111981 (PMC4222958; doi:10.1371/journal.pone.0111981)
Supplement: Table S1 — Scale for Quality Assessment. (DOC) [file pone.0111981.s001.doc]

**Table S1：Scale for Quality Assessment**

| **Quality parameters** | **Score** |
| --- | --- |
| Representativeness of case |  |
| Selected from population cancer registry | 2 |
| Selected from hospital | 1 |
| No method of selection described | 0 |
| Representativeness of control |  |
| Population-based or healthy volunteers | 3 |
| Blood donors | 2 |
| Hospital-based (cancer-free patients) | 1 |
| Not described | 0 |
| Ascertainment of glioma |  |
| Histopathologic confirmation | 2 |
| Diagnosis of glioma by patient medical record or by patient history | 1 |
| Not described | 0 |
| Genotyping examination |  |
| Genotyping done under “blinded” condition | 1 |
| Unblinded or not mentioned | 0 |
| Sample size (total number of cases and controls) |  |
| Larger than 200 | 2 |
| Larger than 100, but less than 200 | 1 |
| Less than 100 | 0 |
| Matching of case and control participants |  |
| Controls matched with cases more than one variable (i.e., age, gender and ethnicity) | 2 |
| Controls matched with cases only one variable (i.e., age, gender or ethnicity) | 1 |
| Not matched or not descried | 0 |
| Total | 12 |

Studies were categorized as ‘‘high quality’’ if the quality score was ≥7; otherwise, studies were categorized as ‘‘low quality’’
