# Supplementary material for: XRCC1 Gene Polymorphisms and Glioma Risk in Chinese Population: A Meta-Analysis
Source: PLoS One. 2014 Nov 6;9(11):e111981. doi: 10.1371/journal.pone.0111981 (PMC4222958; doi:10.1371/journal.pone.0111981)
Supplement: Table S2 — Quality score assessment results. (DOC) [file pone.0111981.s002.doc]

**Table S2**. Quality score assessment results

| First Author | Year | Representativeness  of case | Representativeness  of control | Ascertainment  of glioma | Genotyping examination | Sample size | Matching of case and control participants | Total Score |
| --- | --- | --- | --- | --- | --- | --- | --- | --- |
| Gao | 2014 | 1 | 1 | 1 | 1 | 2 | 2 | 8 |
| Xu | 2013 | 1 | 1 | 2 | 1 | 2 | 2 | 9 |
| Pan | 2013 | 1 | 1 | 2 | 1 | 2 | 2 | 9 |
| Luo | 2013 | 1 | 1 | 1 | 0 | 2 | 2 | 7 |
| Liu | 2012 | 1 | 1 | 2 | 0 | 2 | 2 | 8 |
| Wang | 2012 | 1 | 1 | 2 | 1 | 2 | 2 | 9 |
| Zhou | 2011 | 1 | 1 | 2 | 0 | 2 | 2 | 8 |
| Hu | 2011 | 1 | 1 | 2 | 0 | 2 | 2 | 8 |
| Liu | 2011 | 1 | 1 | 0 | 0 | 1 | 2 | 5 |
